# A delay in diagnosis: thrombotic thrombocytopenia purpura occurring in systemic lupus erythematous

**DOI:** 10.11604/pamj.2019.34.103.20524

**Published:** 2019-10-21

**Authors:** Zoya Adam, Ahmed Sokwala, Jasmit Shah, Sayed Karar Ali

**Affiliations:** 1 Department of Internal Medicine, The Aga Khan University, Nairobi, Kenya

**Keywords:** Thrombotic thrombocytopenia purpura, systemic lupus erythematous, diagnostic challenge

## Abstract

Thrombotic thrombocytopenia purpura (TTP) in the background of systemic lupus erythematous (SLE) remains rare with an incidence of about 2%. Both conditions have overlapping features and can thus be difficult to differentiate and diagnose. A careful review of the peripheral blood smear remain essential often providing many clues. The diagnosis of TTP is a medical emergency and therapy should be instituted immediately. We present one such challenging case where a delay in diagnosis due to limited resources could have proven fatal for our patient.

## Introduction

Thrombotic thrombocytopenia purpura (TTP), is a rare life threatening disease presenting with microangiopathic haemolytic anaemia, thrombocytopenia, renal abnormalities, neurological abnormalities and a fever. However, the urgency for treatment of patients with plasma exchange has resulted in a change in the diagnostic criteria. It has been revised from the earlier classic pentad, found in only 5% of cases, to the current dyad of thrombocytopenia and microangiopathic hemolytic anaemia, with no clinically apparent alternative explanation for thrombocytopenia and anaemia [1]. TTP occurring in the background of systemic lupus erythematous (SLE), remains rare. The incidence of TTP in SLE is thought to be approximately 2% [2]. The pathophysiologic feature of TTP has been described as severe deficiency of von Willebrand Factor (vWF) cleaving metalloproteinase (ADAMTS-13), which normally cleaves the unusually large vWF into smaller and less adhesive vWF moiety. This deficiency is thought to be possibly secondary to the presence of an IgG antibody inhibiting ADAMTS-13 activity, inhibition that finally allows the presence of units of unusually large vWF which is responsible for the microvascular thrombosis, hemolysis, and thrombocytopenia [3]. TTP is difficult to differentiate from a flare of SLE because of overlapping features. Both can present with haemolytic anaemia, thrombocytopenia, fevers, renal and neurological dysfunction, often complicating the diagnosis. The haemolytic anaemia in TTP is microangiopathic while in a flare of SLE autoimmune haemolytic anaemia is the commonest cause. In the background of SLE, a number of disease entities can cause microangiopathic haemolytic anaemia and thrombocytopenia including antiphospholipid syndrome, disseminated intravascular coagulation, malignant hypertension, systemic vasculitis as well as a HELLP syndrome in any female of child bearing age [4]. The mainstay of treatment of TTP even in the background of SLE remains plasma exchange [5]. Corticosteroids are used initially to achieve relatively rapid immunosuppression. There is some prospective evidence that higher doses of methylprednisolone (10 mg/kg/day) are more effective than lower doses (1 mg/kg/day) [6]. Rituximab is effective in patients who have failed to respond to plasma exchange and steroids [7].

## Patient and observation

A 40-year-old female of Asian origin, known to have SLE diagnosed three years ago, presented to our institution with a three-day history of jaundice, gross hematuria and bleeding from her gums. Apart from generalized weakness and intermittent headaches, the patient denied any other symptoms including abdominal pain, diarrhea, vomiting, dysuria, frequency, fever or chills, neck stiffness, photophobia or seizures. She denied any travel history especially to areas endemic of malaria. She also denied any prior or current history of easy bruising or bleeding tendencies. Her past medical history was significant for systemic lupus erythematous with a flare treated with high dose steroid one year ago. Her current medications included: prednisolone 2.5mg once a day. She was a mother of two healthy children and was currently not on any contraception. Her menstrual cycle was regular and denied any previous miscarriages. She denied any alcohol or tobacco history and was active with daily household chores. Her physical exam was significant for a middle aged Asian female in no apparent distress. She had obvious palmar and conjunctival pallor, as well as scleral jaundice. There was no presence of lymphadenopathy or edema. Examination of her skin showed an echymotic lesion measuring 2x3 cm on her right arm. Her cardiovascular, abdominal and neurological exams were unremarkable. Laboratory findings revealed a normal white blood cell count, a normocytic normochromic anemia (Hb-10.1g/dl, MCV-82, MCH-28) and a thrombocytopenia of 7x109/L. The reticulocyte count was elevated (9.4%) and so was the level of lactate dehydrogenase (1554 iu/L). Urea, electrolytes and creatinine were within normal, however urine microscopy revealed proteinuria 2+, blood 3+, and red blood cells 2+. Liver function tests showed an indirect hyperbilirubinemia (total bilirubin 102μmol/litre and direct bilirubin 28μmol/litre). The other parameters of her liver function tests were within normal. The coagulation profile that included APTT and PT were within normal limits. A C reactive protein was elevated at 17.5mg/L while C3 and C4 levels were low. The patient's septic screen did not show any foci of infection.

**Figure 1:**
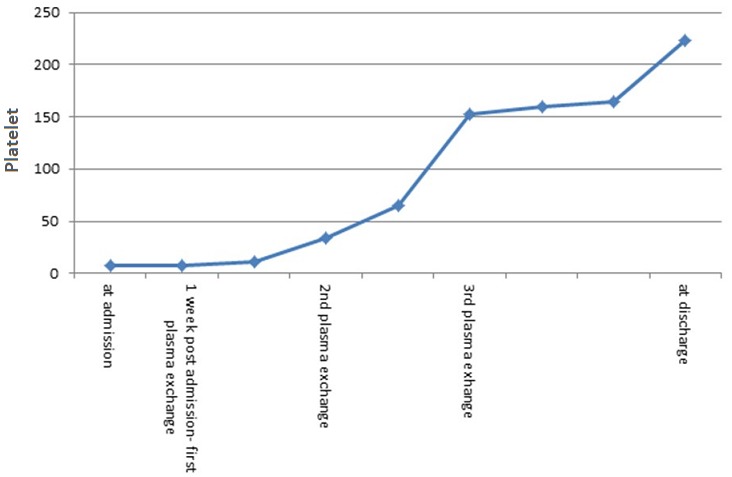
Platelet count from admission to discharge

Her Chest x-ray was normal and her urine and blood cultures remained negative. A procalcitonin was within normal and the malaria parasite smear was negative. A HIV, Hepatitis B and C panels were negative. In view of her normocytic normochromic anaemia with an elevated reticulocyte count and LDH, as well as the indirect hyperbilirubinemia, a haemolytic anaemia was suspected. The low C3 and C4 levels were thought to be as a result of a flare of SLE that had led to autoimmune destruction of red blood cells as well as platelets. The patient was started on pulse doses of methylprednisolone (1 gram intravenously per day) for three days and then continued on oral prednisolone at 1mg/kg (60mg/day). Meanwhile a peripheral blood film and a direct Coombs test were ordered to confirm the diagnosis of autoimmune haemolytic anaemia. Over the next 5 days, the patient's haemoglobin and thrombocytopenia continued to drop, despite the steroids. The indirect hyperbilirubinemia and LDH levels remained high indicating persistent on-going hemolysis. At the end of 5 days the direct Coombs test result was negative and a peripheral blood film report revealed schistocytes suggestive of a microangiopathic haemolytic anaemia (MAHA), not an autoimmune haemolytic anaemia. An antiphospholipid panel was negative ruling out the possibility of antiphospholipid syndrome. A pregnancy test was also negative ruling out the possibility of HELLP syndrome causing MAHA. Hence, a diagnosis of TTP was then made in view of the patient exhibiting 3 out of 5 features of the classic pentad (MAHA, thrombocytopenia and headaches). Plasma exchange was immediately initiated at a dose of 1 volume on alternate days and the steroids at 1mg/kg were continued. Alternate day plasma exchange as opposed to the recommended daily plasma exchange was pursued due to the difficulty in obtaining an adequate number of fresh frozen plasma units. After three sessions of plasma exchange, the patient's platelet count increased to 152x109 /L, her LDH level normalized and her headaches markedly improved. The platelet count continued to progressively rise. On discharge, four weeks later, the patient's hemoglobin count was 10.8g/dl, platelet count of 228x109/ L and she had a normal total bilirubin. Her steroids were gradually tapered and she was scheduled to follow in our outpatient clinic. Figure 1 represents the platelet count from admission to discharge of the patient.

## Discussion

The initial diagnosis in our patient was thought to be a flare of SLE leading to the autoimmune mediated destruction of red blood cells and platelets. However, the peripheral blood smear was remarkable for schistocytes and not spherocytes. In addition, our patient had a normocytic normochromic anaemia with an elevated reticulocyte count, a negative coombs test, an elevated lactate dehydrogenase and an indirect hyperbilirubinemia, suggestive of non-immune mediated hemolysis. Microangiopathic haemolytic anemia in a patient with SLE can have a number of causes including: disseminated intravascular coagulation (DIC), malignant hypertension, catastrophic antiphospholipid syndrome, Thrombotic thrombocytopenic purpura [4]. In this patient a coagulation profile (APTT, PT) was normal ruling out DIC. An antiphospholipid panel was also negative. The blood pressure in our patient throughout her hospital course remained within normal ranges, ruling out the possibility of malignant hypertension. TTP occurring in patients with SLE can be difficult to diagnose because of overlapping features of the two disorders and the presence of other potentially concomitant thrombotic microangiopathies. Both TTP and a flare of SLE can present with haemolytic anaemia, thrombocytopenia, fevers, renal and neurologic manifestations. Hence a peripheral blood film is essential as the finding of ≥ 1% schistocytes favours a diagnosis of TTP [5]. A negative coombs test favours TTP, but a positive Coombs test does not rule out TTP. TTP in our patient was diagnosed on the basis of a peripheral blood film that showed ≥ 1% schistocytes indicative of a microangiopathic anaemia, thrombocytopenia, as well as neurologic (headache) and renal (proteinuria hematuria on urinalysis) manifestations.

Even though not sensitive or specific, a severe deficiency of ADAMTS-13 activity and the presence of ADAMTS-13 inhibitors in the appropriate clinical setting can confirm the presence of TTP [8]. Normal ADAMTS-13 activity should clue one into looking at other causes of MAHA and low platelets [9]. Interestingly, in patient with SLE, especially with a positive anti-dsDNA, and concomitant TTP, less than 10% showed ADAMTS-13 activity [8]. In addition, the decreased level of ADAMTS-13 activity correlated with increased SLE related tissue damage [10]. The diagnosis of TTP is a medical emergency with plasma exchange being the mainstay of treatment. Plasma exchange should be started immediately and has shown to decrease mortality for an estimated 90% to less than 10% [11]. Hence now the dyad of microangiopathic haemolytic anaemia and thrombocytopenia with no clinically apparent explanation should be treated as TTP [1]. Unfortunately, in our patient's case the diagnosis of TTP was delayed by a week due to the delay in the report of the peripheral blood film. Plasma exchange is usually given concomitantly with steroid [4]. Once the platelets have normalized for 48 hours, the plasma exchange can be halted and steroid gradually tapered [4,12]. Cyclophosphamide and rituximab (anti-CD 20) have been reportedly used as a salvage treatment in refractory TTP [12,13]. This case report demonstrates that not all hemolytic anaemia in SLE is immune mediated. Peripheral blood film is mandatory to evaluate anemias even though it is often over looked. Microangiopathic haemolytic anaemia in the background of SLE can have a number of etiologies including TTP. Diagnosing TTP in the background of SLE poses a challenge. Diagnosis and appropriate management was possible in this patient albeit a week after admission. The patient responded well and was discharged after complete recovery.

## Conclusion

Hemolytic anemia and thrombocytopenia must always raise the suspicion on TTP especially in the appropriate clinical setting. SLE and TTP share similar haematological manifestations and the presence of both can often be lethal. Early diagnosis and treatment remain key in ensuring positive outcomes.

## Competing interests

The authors declare no competing interests.
